# Mesenchymal progenitor cells from non-inflamed versus inflamed synovium post-ACL injury present with distinct phenotypes and cartilage regeneration capacity

**DOI:** 10.1186/s13287-023-03396-3

**Published:** 2023-06-25

**Authors:** Roman J. Krawetz, Leila Larijani, Jessica May Corpuz, Nicoletta Ninkovic, Nabangshu Das, Alexandra Olsen, Nicholas Mohtadi, Alexander Rezansoff, Antoine Dufour

**Affiliations:** 1 McCaig Institute for Bone and Joint Health, Faculty of Medicine, University of Calgary, 3330 Hospital Drive NW, Calgary, AB T2N 4N1 Canada; 2 Department Cell Biology and Anatomy, University of Calgary, Calgary, AB Canada; 3 Department of Surgery, University of Calgary, Calgary, AB Canada; 4 Department of Biomedical Engineering, University of Calgary, Calgary, AB Canada; 5 Sport Medicine Centre, Faculty of Kinesiology, University of Calgary, Calgary, AB Canada; 6 Department of Physiology and Pharmacology, University of Calgary, Calgary, AB Canada

**Keywords:** Mesenchymal progenitor cells, Osteoarthritis, Inflammation, ACL injury

## Abstract

**Background:**

Osteoarthritis (OA) is a chronic debilitating disease impacting a significant percentage of the global population. While there are numerous surgical and non-invasive interventions that can postpone joint replacement, there are no current treatments which can reverse the joint damage occurring during the pathogenesis of the disease. While many groups are investigating the use of stem cell therapies in the treatment of OA, we still don’t have a clear understanding of the role of these cells in the body, including heterogeneity of tissue resident adult mesenchymal progenitor cells (MPCs).

**Methods:**

In the current study, we examined MPCs from the synovium and individuals with or without a traumatic knee joint injury and explored the chondrogenic differentiation capacity of these MPCs in vitro and in vivo.

**Results:**

We found that there is heterogeneity of MPCs with the adult synovium and distinct sub-populations of MPCs and the abundancy of these sub-populations change with joint injury. Furthermore, only some of these sub-populations have the ability to effect cartilage repair in vivo. Using an unbiased proteomics approach, we were able to identify cell surface markers that identify this pro-chondrogenic MPC population in normal and injured joints, specifically CD82^Low^CD59^+^ synovial MPCs have robust cartilage regenerative properties in vivo.

**Conclusions:**

The results of this study clearly show that cells within the adult human joint can impact cartilage repair and that these sub-populations exist within joints that have undergone a traumatic joint injury. Therefore, these populations can be exploited for the treatment of cartilage injuries and OA in future clinical trials.

**Supplementary Information:**

The online version contains supplementary material available at 10.1186/s13287-023-03396-3.

## Background

Osteoarthritis (OA) is a painful and debilitating disease which has no known “cure” and there are no current treatments which can predictably slow, stop or reverse its progression [1, 2]. Our goal is to develop methods for non-surgical therapeutic management of OA. Based on recent development including pre-clinical [3, 4] and clinical [5–8] studies examining the use of adult stem/progenitor cell populations for the treatment of OA, we propose it is essential to understand the biology of endogenous mesenchymal progenitor cell (MPC) populations within the joint. Specifically, we need to determine if these cells can contribute to endogenous repair of damaged joint tissues and if so, why this does not occur in patients suffering from cartilage injury and/or OA. Therefore, in the current study we focused on adult mesenchymal progenitor cells derived from the synovium.

We have previously demonstrated that MPCs derived from joint tissues (synovium [9, 10] or synovial fluid [11, 12]; knee or hip [13]) from individuals with no history of joint disease/injury, e.g., normal joints; have the capacity to generate cartilage-like tissue in vitro and in vivo [14]. This implies that synovial MPCs under ‘ideal’ conditions can become cartilage and therefore may be uniquely positioned to repair cartilage defects in vivo [15, 16]. However, we have also observed that MPCs derived from the OA synovium (late-stage samples from joints undergoing replacement; knee and hip) demonstrate decreased chondrogenic potential in vitro [9, 11, 13]. Yet, it is also important to consider that MPCs do not necessarily need to differentiate into new tissue to contribute to repair. A growing body of literature has clearly demonstrated that these cell populations can regulate the inflammatory response and/or empower terminally differentiated cell populations to effect repair [17–20]. Interestingly, we and others have shown that MPC phenotype can also be dramatically impacted by the local microenvironment [21, 22], even going as far as mitigating the differentiation potential of the cells [9, 11]. This suggests that the disease state itself has the potential to modify the cells behavior. We have previously shown that the synovial MPCs can be permanently modified by the OA joint environment as they do not regain the same capacities as normal synovial MPCs once removed from the joint and cultured under standard conditions [11]. When these results are considered in the context of tissue resident MPCs within the joint and their ability to migrate to areas of cartilage damage [23], this strongly suggests that the inflammatory environment present in the injured and/or OA joint modifies cell phenotype to the point where MPCs are mobilized and migrate to the defect but cannot affect cartilage repair (directly or indirectly). At this threshold point any further endogenous synovial MPC response would likely be completely ineffective in cartilage repair and the balance swings dramatically to cartilage degeneration [24].

While there is a growing body of literature on joint resident MPCs in OA joints, we are lacking information on synovial MPCs from patients that have recently suffered an intra-articular injury. This is a significant gap in our knowledge since we need to identify if synovial MPCs from recently injured joints have also lost the potential for cartilage repair. There are several reasons to consider the recently injured joint: (1) Major injuries, for example to the knee, are very common in young people; (2) Knee injury is a well-recognized risk factor for the development of post-traumatic osteoarthritis; (3) Cell therapies (synovial MPCs) have the potential to slow/stop/reverse the onset of OA; (4) If autogenous synovial MPCs from injured joints lack chondrogenic potential then allogeneic cell transplants from healthy, normal joints should be the focus of future research.

## Methods

### Ethics statement

Human: Informed consent to participate was obtained by written agreement. The study protocol was approved by the University of Calgary Research Ethics Board (University of Calgary ethics # REB16-1262, Collection of Synovial Fluid and Tissues During Arthroscopic Knee Surgery, Approval Date: August 12, 2016, Approval #: N/A). The study was carried out in accordance with the declaration of Helsinki.

Animal: For in vivo experiments, equal numbers of male and female 10-week-old mice were used [14]. Mice were randomized across treatment groups. This study was carried out in accordance with the recommendations in the Canadian Council on Animal Care Guidelines. Animal protocols and surgical procedures were approved by the University of Calgary Animal Care Committee (Ethics # AC20-0042, Stem cell tracking in mouse knee joints, Approval Date: May 25, 2020, Approval #: N/A). All surgery was performed under isoflurane anesthesia. Number of repeats is specified in each figure legend. Our manuscript reporting adheres to the ARRIVE guidelines.

### Study participants

A total of 19 human subjects with an acute intra-articular anterior cruciate ligament (ACL) joint injury (15 males, 4 females, ACL tear ± meniscal/chondral damage, age 18–28, and body mass index (BMI) < 30) who were scheduled for ACL reconstructive surgery were included in the current study. At the time of surgery, arthroscopic synovial biopsies were obtained from 3 separate areas within the joint (anterior, medial and lateral compartments).

Normal control tissue samples (*n* = 5) were obtained from the Southern Alberta Tissue Donation Program (3 males, 2 females, age 18–41, and BMI < 30). Criteria for control cadaveric donations were, no history of arthritis, joint injury or surgery (including visual inspection of the cartilage surfaces during recovery), no prescription anti-inflammatory medications, no co-morbidities (such as diabetes/cancer), and availability within 4 h of death [9, 10, 14, 25]. A minimum of 4 synovial biopsies (total) were taken by the recovery team from the medial and lateral compartments of the joint adjacent to the capsule.

Each 3 mm^2^ synovial biopsy was be divided in half. One half was processed for immunohistochemistry (IHC) and the other was used to derive synovial MPCs.

### Synovial MPC culture

An outgrowth method was utilized to culture synovial MPCs [9, 10, 14]. Upon receipt of synovium, each tissue explant was minced and seeded in a 24-well culture plate and incubated at 37 °C and 5% carbon dioxide (CO_2_) with 1 mL of MesenCult™ (Stemcell Technologies) culture media added. Within 11 days post-seeding, outgrown cells were adherent to the plastic and reached 30–40% confluency. At this point, cells were gently dissociated via mechanical stimulation and seeded into a T-25 cell culture flask. Media was changed every 3 days. After cells reached 70% confluency, the cells were washed, resuspended, and subjected to magnetic-activated cell sorting (MACS) purification entailing hematopoietic lineage depletion (FCGR3A, CD19, CD3E, NCAM1, CD14, GYPA, FCGR3B, ITGA2B)(BD Biosciences). Purified cells were then expanded in T-75 flasks. Media was changed every three days and cells were passaged when 70–80% confluency was reached. Cells passaged a maximum of 3 times were used for flow cytometry phenotyping, multipotent differentiation analysis and proteomics.

### Flow cytometry and cell sorting

Synovial MPCs were fixed in methanol for 10 min on ice. After PBS washing, cells were blocked for 30 min at 37 °C with 3% BSA. They were then incubated away from light for 1 h with fluorescent antibodies for CD105 (Clone # 266), CD90 (Clone # 5E10), CD73 (Clone # AD2), CD44 (Clone # G44-26), CD45 (Clone # HI30), CD11b (Clone # D12) (all BD) prior to flow cytometric analysis on an Invitrogen Attune® Acoustic Focusing Cytometer.

To obtain the purified sub-populations of MPCs, cells were stained with CD59 (Clone # p282), CD99 (Clone # hec2) and CD82 (Clone # ASL-24) (All Biolegend) and then underwent fluorescent activated cell sorting (FACS) on a BD FACS Aria Fusion (BD Biosciences). Dead cells (FVS510^+^) were excluded. The remaining cells were sorted based on CD59^+^ or CD82^High^ expression using a 100 μM sort nozzle and low flow rate (45% of system maximum) to reduce the pressure on the cells. The resultant cell populations were placed back into culture and expanded until a we obtained sufficient number of cells to undertake the downstream experiments.

### Differentiation

The MPCs underwent multi-lineage differentiation analysis to determine their osteo/chondro/adipo-genic capacity.

Osteogenesis: For each replicate, 5 × 10^5^ cells were seeded into each well in a 24-well plate and then placed into DMEM/F-12 media that contained Dexamethasone (final concentration (FC): 100. nM) (Sigma), L-Ascorbic Acid (FC: 50 μg/mL) (Sigma), β-Glycerolphosphate (FC: 10. mM) (Sigma).

Adipogenesis: For each replicate, 5 × 10^5^ cells were seeded into each well in a 24-well plate and then placed into DMEM/F-12 media that contained Dexamethasone (FC: 1 μM) (Sigma), Insulin (FC: 10 μM) (Sigma), Indomethacin (FC: 200 μM) (Sigma), and Isobutylmethylxanthine (FC: 500 μM) (Sigma).

Chondrogenesis: For each replicate, 5 × 10^5^ cells were pelleted through centrifugation and placed into DMEM/F-12 media that contained Dexamethasone (FC: 10 nM) (Sigma), L-Ascorbic Acid (FC: 50 μg/mL) (Sigma), MEM Non-Essential Amino Acids (FC: 1%) (MEM-NEAA Gibco), Transforming growth factor (TGF)-β3 (FC: 10 ng/mL) (Peprotech), Bone morphogenetic protein (BMP)-2 (FC: 500 ng/mL) (Peprotech), insulin transferrin selenium (FC: 1%) (Lonza- BioWhittaker), and sodium pyruvate (FC: 1%) (ThermoFisher). Media was adjusted to neutral pH (7.0–7.6).

After 21 days, differentiation was assayed using reverse transcriptase quantitative polymerase chain reaction (RT-qPCR).

### RT-qPCR

mRNA was isolated using the TRIzol reagent protocol (ThermoFisher) following the manufactures instructions with the addition of glycogen solution (Amresco) to increase the yield of mRNA. Chondrogenic cultures alone went through an additional spin column step (OMEGA bio-tek E.Z.N.A. Total RNA Kit I) to remove additional ECM proteins which could potentially interfere with downstream applications. For first strand synthesis, mRNA was then added cDNA Master Mix (High-Capacity cDNA kit, Applied Biosystems) following the manufactures instructions. The cDNA was stored at − 20 °C until use.

For osteogenesis, gene expression of Osterix (*Sp7*) (Probe set # Hs01866874_s1) and *Runx2* (Probe set # Hs01047973_m1) were quantified. For adipogenesis, *Adipoq* (Probe set # Hs00977214_m1) was quantified. For chondrogenesis, *Sox9* (Probe set # Hs00165814_m1), *Acan* (Probe set # Hs00153936_m1) and *Col2a* (Probe set # Hs00264051_m1) were quantified. Ribosomal 18S (Probe set # Hs99999901_s1) was employed as a housekeeping gene. All TaqMan Gene Expression Assays were obtained from Applied Biosystems (ABI). Three replicates were run per sample and all samples were run on an ABI Quantstudio6 (Applied Biosystems) Resulting threshold (Ct) values were analyzed using the ΔΔCt method against 18S endogenous control and undifferentiated cells as the reference sample.

### Cartilage Injury Model

Full thickness cartilage defects (FTCD) [3, 26–29] were created on the tibial plateau in NOD *scid* gamma (NSG) mice. Animals were administered an intraperitoneal injection of buprenorphine (0.05 mg/kg) prior to surgery and anaesthetized under isoflurane (Baxter) anesthesia (1.5% v/v O2) for the duration of the surgical procedure. Briefly, a small incision was made on the medial side of the left knee. A depth stopped 26G needle (diameter = 450 µm, length to stopper = 600 µm) was used to gently displace the patella and expose the trochlear groove of the femur. A slight pressure, combined with a twisting motion, was applied at the contact with the trochlear groove to make a circular wound that penetrated no farther than 600 µm into the underlying subchondral bone. The needle was gently removed, and the skin closed with a sterile wound clip after the FTCD was made. The mice were randomly assigned to a group and intraarticularly injected 1-week post-injury with 100,000 human synovial MPCs in 2 µL of sterile saline [14]. The mice were euthanized 4 weeks post-injection by CO_2_ asphyxiation, followed by cervical dislocation as outlined in the approved animal protocol.

### Histology

Slides were stained routinely with hematoxylin and eosin (H&E) and by immunohistochemistry (IHC). Subjective scoring of synovitis was performed on H&E stained synovium using the scale of Krenn et al. [30]. Briefly, three features of synovitis: enlargement of lining cell layer, cellular density of synovial stroma and leukocytic infiltrate were evaluated from 0—absent, to 3—strong and each feature was graded separately. The sum provided the synovitis score, which was interpreted as: 0–1, no synovitis; 2–4, low-grade synovitis; 5–9, high-grade synovitis. Grading was performed by three individuals in total, 2 were blinded.

Human synovial sections (10 μm) were deparaffinized in CitriSolv (Fisher Scientific; Fairlawn, NJ, USA) and rehydrated through a series of graded ethanol to distilled water steps. Antigen retrieval (10 mM sodium citrate, pH 6.0, Sigma-Aldrich) and blocking (1:500 dilution; 100 μL goat serum: 50 mL TRIS-buffered saline, 0.1% Tween 20 (TBST) for 1 h), steps were performed prior to going through sequential wash (TBST) and the application of primary antibody. Antibodies conjugated to fluorophores included: CD99, CD82 and CD52 (All Biolegend). All slides were mounted using EverBrite™ Hardset Mounting Medium with DAPI (Biotium). Slides were imaged using a Plan-Apochromat objective on an Axio Scan.Z1 Slide Scanner microscope (Carl Zeiss).

Mouse knee joints were fixed in 10% neutral buffered formamide (NBF) (Fisherbrand) for 7 days and decalcified in 10% ethylenediaminetetraacetic (EDTA) for 14 days. Samples underwent tissue processing, were embedded in paraffin wax, and then sectioned at 10 µm. Histological analysis was then conducted on the knee sections. Samples were deparaffinized in Citrosolv (Decon Laboratories), and then rehydrated in a series of ethanol washes with decreasing concentration. For histological analysis, samples were stained with Safranin-O/Fast-green and graded based on a previously published scoring system [29]. The parameters of the scoring system include cell morphology (0–4), matrix staining (0–3), surface regularity (0–3), thickness of cartilage (0–2) and integration with native cartilage (0–2). On this scale, uninjured native articular cartilage is 14, while the absence of cartilage is 0.

Immunohistochemistry analysis was performed on the mouse knee sections. Antigen-retrieval was achieved using 10 mM sodium citrate (pH 6.0), and non-specific blocking was prevented using goat-serum (1:500 dilution in TBST). Human nuclear antigen (HNA; Clone # 235-1, Abcam) or Collagen 2 (Col2; Clone # II-II6B3, DSHB) was applied to the sections and incubated overnight. For the Col2 staining an additional hyaluronidase (Sigma) treatment step was added. Anti-rabbit secondary antibody AF647 (1:100, Biolegend) was applied the next day. Secondary controls were also performed, where only secondary antibody was applied to the sections (no primary antibody). All slides were mounted using EverBrite™ Hardset Mounting Medium with 4′,6-diamidino-2-phenylindole (DAPI, Biotium). Slides were imaged using a Plan-Apochromat objective on an Axio Scan.Z1 Slide Scanner microscope (Carl Zeiss).

### Tissue cytometry

For quantitative analysis, the area of interest was acquired as digital greyscale images. Cells of a given phenotype were identified and quantitated using the TissueQuest software (TissueGnostics), with cut-off values determined relative to the negative controls (non-stained and secondary alone controls). Gating and quantification of single/double positive cells were undertaken using these thresholds [31].

### High performance liquid chromatography (HPLC) and Tandem mass spectrometry (MS/MS)

All liquid chromatography and mass spectrometry experiments were carried out by the Southern Alberta Mass Spectrometry (SAMS) core facility at the University of Calgary, Canada. Analysis was performed on an Orbitrap Fusion Lumos Tribrid mass spectrometer (Thermo Scientific) operated with Xcalibur (version 4.0.21.10) and coupled to a Thermo Scientific Easy-nLC (nanoflow Liquid Chromatography) 1200 system. Tryptic peptides (2 μg) were loaded onto a C18 trap (75 μm × 2 cm; Acclaim PepMap 100, P/N 164946; ThermoScientific) at a flow rate of 2 μl/min of solvent A (0.1% formic acid and 3% acetonitrile in LC–MS grade water). Peptides were eluted using a 120 min gradient from 5 to 40% (5% to 28% in 105 min followed by an increase to 40% B in 15 min) of solvent B (0.1% formic acid in 80% LC–MS grade acetonitrile) at a flow rate of 0.3 μL/min and separated on a C18 analytical column (75 um × 50 cm; PepMap RSLC C18; P/N ES803; Thermo Scientific). Peptides were then electrosprayed using 2.3 kV voltage into the ion transfer tube (300 °C) of the Orbitrap Lumos operating in positive mode. The Orbitrap first performed a full MS scan at a resolution of 120,000 Full Width at Half Maximum (FWHM) to detect the precursor ion having a *m*/*z* between 375 and 1,575 and a + 2 to + 7 charge. The Orbitrap AGC (Auto Gain Control) and the maximum injection time were set at 4 × 10^5^ and 50 ms, respectively. The Orbitrap was operated using the top speed mode with a 3 s cycle time for precursor selection. The most intense precursor ions presenting a peptidic isotopic profile and having an intensity threshold of at least 5,000 were isolated using the quadrupole and fragmented with higher collisional energy (30% collision energy) in the ion routing multipole. The fragment ions (MS^2^) were analyzed in the ion trap at a rapid scan rate. The AGC and the maximum injection time were set at 1 × 10^4^ and 35 ms, respectively, for the ion trap. Dynamic exclusion was enabled for 45 s to avoid of the acquisition of same precursor ion having a similar m/z (plus or minus 10 ppm).

### Proteomic data and bioinformatics analysis

Data are available via ProteomeXchange with identifier PXD026787. Spectral data were matched to peptide sequences in the human UniProt protein database using the Andromeda algorithm as implemented in the MaxQuant [32, 33] software package v.1.6.10.23, at a peptide-spectrum match FDR of < 0.01. Search parameters included a mass tolerance of 20 p.p.m. for the parent ion, 0.5 Da for the fragment ion, carbamidomethylation of cysteine residues (+ 57.021464 Da), variable N-terminal modification by acetylation (+ 42.010565 Da), and variable methionine oxidation (+ 15.994915 Da). N-terminal and lysine heavy (+ 34.063116 Da) and light (+ 28.031300 Da) dimethylation were defined as labels for relative quantification. The cleavage site specificity was set to Trypsin/P for the proteomics data, with up to two missed cleavages allowed. Significant outlier cutoff values were determined after log(2) transformation by boxplot-and-whiskers analysis using the BoxPlotR tool [34].

### Statistics

The RT-qPCR data were analyzed using GraphPad Prism 7 (GraphPad Software). The data is reported as ± standard deviation (SD). Statistical analysis was performed with a paired t-test since the undifferentiated controls (or ± CD populations) for each experiment performed are derived from the same parental cells as the differentiated cells (or mean). An alpha value of *p* < 0.05 was regarded as statistically significant.

## Results

### Differences in synovial inflammation

**Fig. 1:**
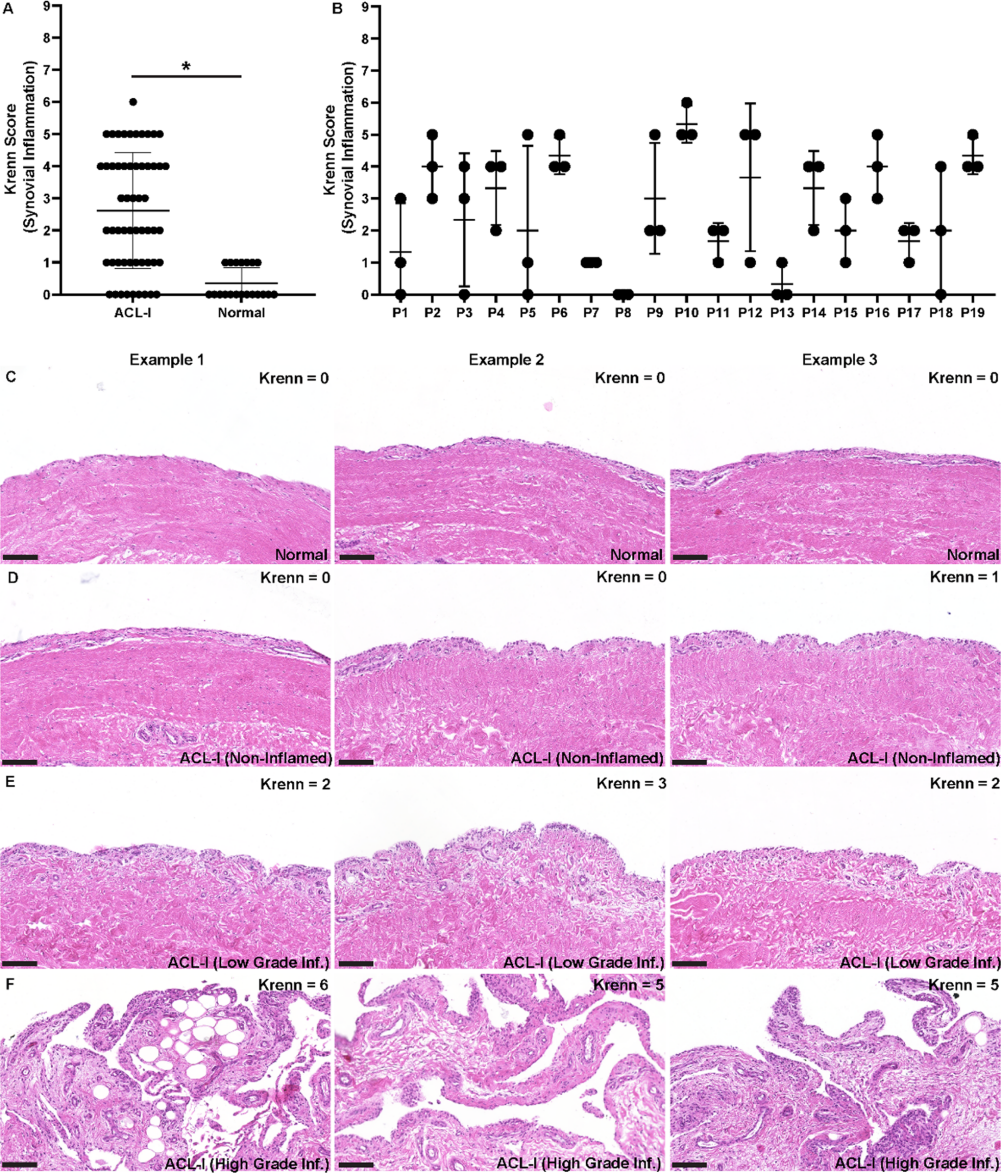
Synovitis within ACL-I and normal joints. Synovitis was graded within ACL-I (*n* = 19, 3 biopsies per individual) and normal knee joints (*n* = 5, 3 biopsies per individual) using the histological grading score developed by Krenn et al. (**A**). Individual biopsies were graded within every joint and heterogeneity was observed between biopsies locations within a given joints and also between patients (**B**). Three representative images of normal (**C**) and ACL-I (**D**–**F**) synovium presenting with no, low-, and high-grade synovitis. Scale bar equal 100 µm. *p < 0.05

Synovial samples from all individuals in the study including ACL injury (ACL-I) and normal control (normal) were graded for synovial inflammation using the system developed by Krenn et al. [30] (Fig. 1). There were significant differences in synovial inflammation between normal and ACL-I biopsies with increased inflammation present in the synovium of ACL-I patients (Fig. 1A). Within the normal samples, there was minimal synovitis observed with little heterogeneity between samples from the same joint or between joints. In ACL-I patients there was a wide range of synovitis score ranging from no synovitis to high grade synovitis, and this level of heterogeneity was even observed within the same joint (Fig. 1B). However, some ACL-I joints had little to no synovitis, while others showed synovitis in all biopsies. Most joints however, presented with a range of synovitis severity between biopsies. Representative H&E images are presented for normal (Fig. 1C) and ACL-I (Fig. 1D–F) biopsies, with differing grades of synovitis shown for the ACL-I patients. Based on these results, we decided to examine synovial MPCs from multiple biopsies locations in 3 ACL-I patients: #1, #3 and #18 in more detail since all of these patients presented with areas of both no and moderate synovitis within the same joint.

### Characterization of MPCs from synovium with low versus high grade synovitis

**Fig. 2:**
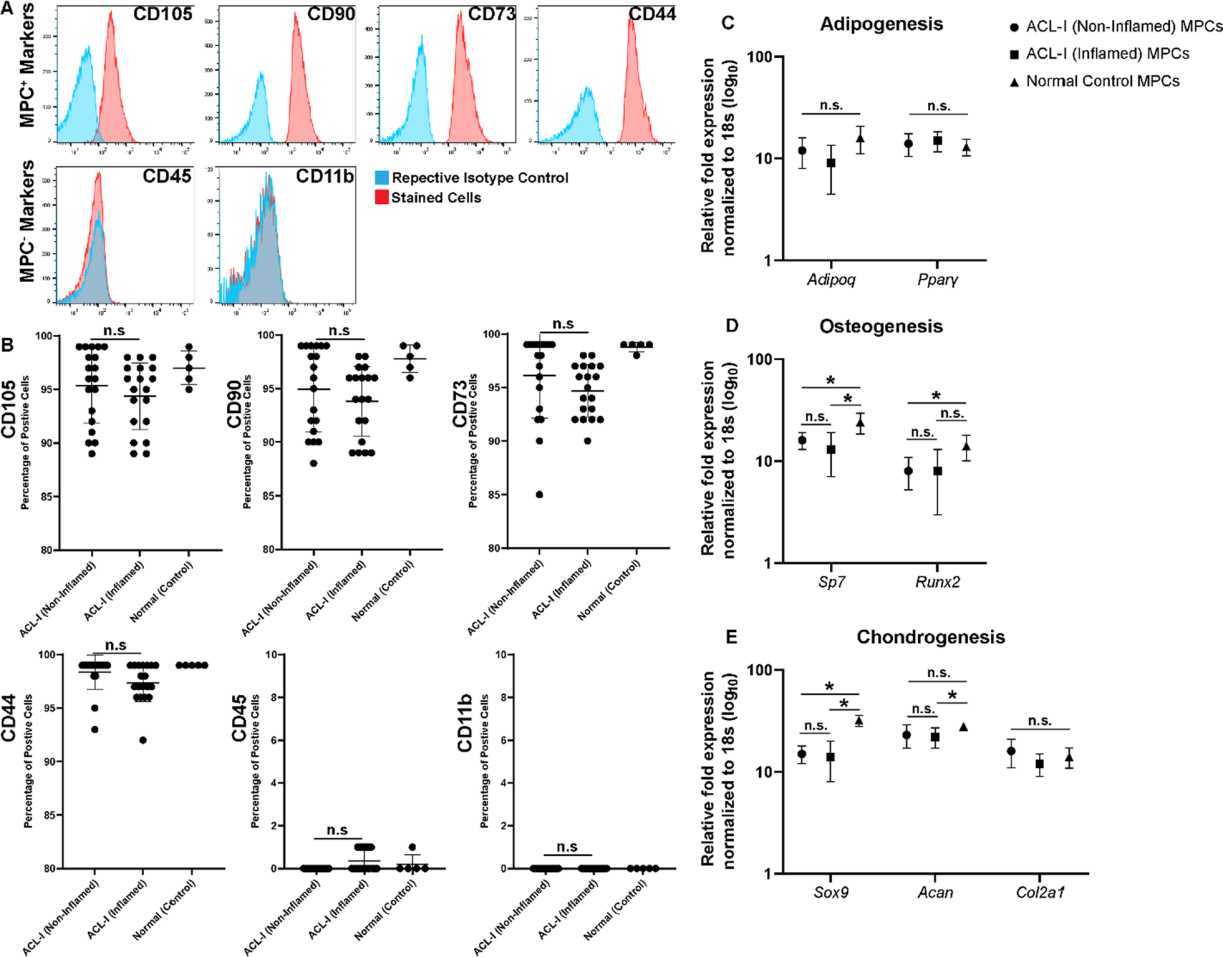
Phenotypic characterization of synovial MPCs. Flow cytometry was employed to quantify the expression of the MPC^+^ (CD105, CD90, CD73, CD44) and MPC^−^ (CD45, CD11b) cell surface markers (**A**). The relative expression of each marker was quantified within each group and compared between groups (**B**). No differences in any marker were observed between ACL-I groups. Adipogenic (**C**), osteogenic (**D**) and chondrogenic (**E**) differentiation was assayed by qPCR expression using lineage specific markers. n.s. = not significant; *p < 0.05

MPCs were derived from normal synovium (*n* = 3) and ACL-I (*n* = 3) patients. In each ACL-I patient, MPCs were derived from one biopsy with a Krenn score equal or less than 1 (termed ACL-I non-inflamed) and one biopsy from a site with a Krenn score equal or greater than 3 (termed ACL-I inflamed). All cells were assayed by flow cytometry for characteristic cell surface markers of MPCs [35] and multipotent differentiation capacity (Fig. 2). MPCs from all groups (normal versus ACL-I) and all grades of synovitis (ACL-I non-inflamed versus ACL-I inflamed) demonstrated robust expression of the typical MPC markers (CD105, CD90, CD73, CD44) and lacked the expression of hematopoietic lineage markers (CD45, CD11b) (Fig. 2A). While there was some level of heterogeneity in the marker expression between the different groups, there were no significant differences in the expression of any marker between ACL-I biopsies (non-inflamed versus inflamed) (Fig. 2B). When the adipogenic, osteogenic and chondrogenic differentiation potential was examined, it was found that all groups of MPCs retained the ability to undergo multipotent differentiation. There were no significant differences in *ADIPOQ* or *PPARγ* gene expression between any groups (Fig. 2C) post-adipogenic induction. Post-osteogenic induction, there was no difference in *SP7* and *RUNX2* expression between ACL-I groups (non-inflamed versus inflamed), however, both groups showed decreased marker expression compared to the normal MPC populations (Fig. 2D). There were also no differences in *SOX9*, *ACAN* or *COL2A1* expression between ACL-I groups (non-inflamed versus inflamed) post-chondrogenic induction (Fig. 2E).

### Xeno-transplantation of synovial MPCs into a murine cartilage injury model

**Fig. 3:**
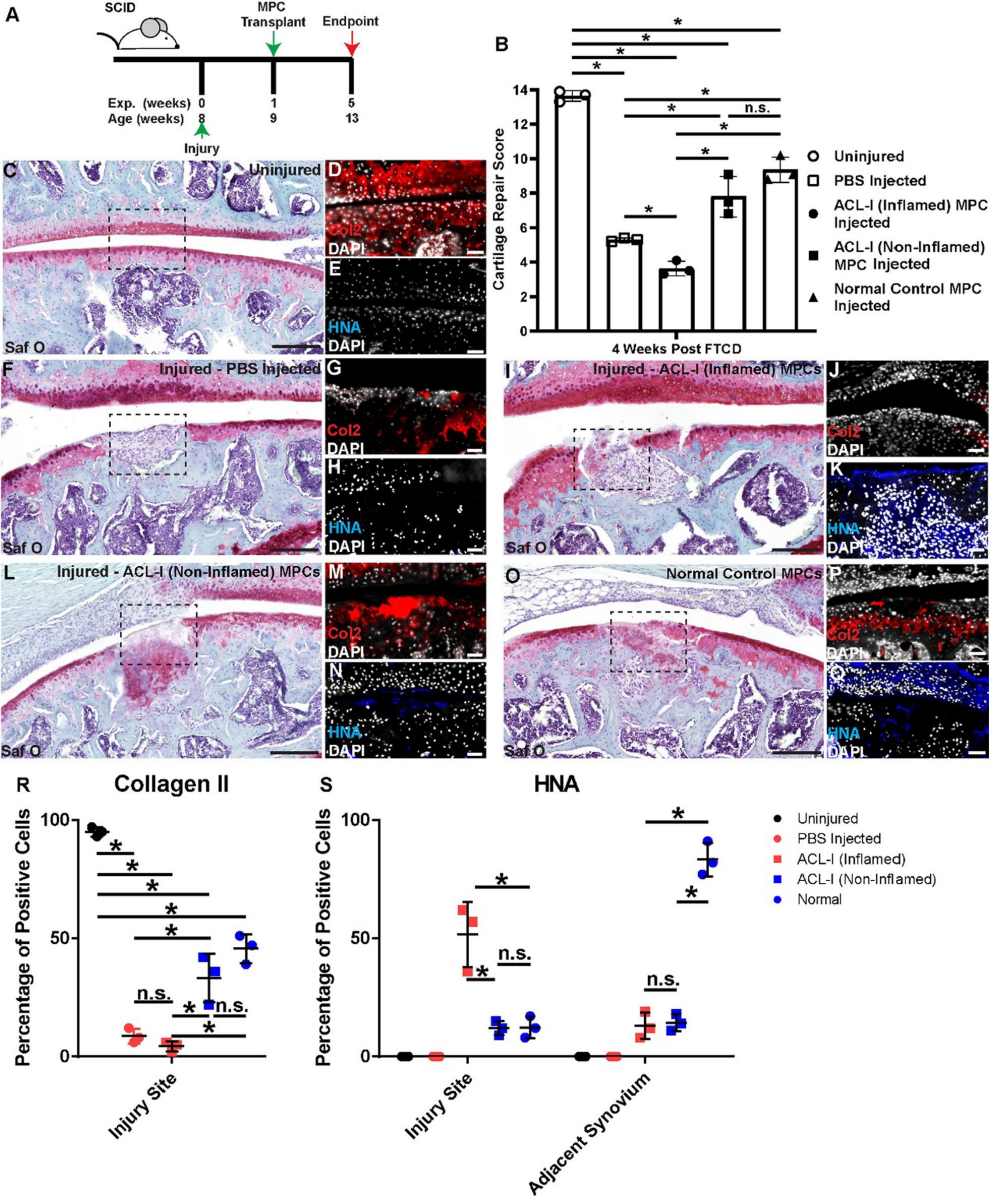
Transplanted human MPCs into mice with a full thickness cartilage defect. Synovial MPCs were injected in NGS mice post-FTCD (**A**). Cartilage repair across all groups was quantified (**B**). Safranin O staining of uninjured (**C**), injured mice injected with saline (**F**) or MPCs from ACL-I (Inflamed, *n* = 3 patients) (**I**), ACL-I (Non-Inflamed, *n* = 3 patients) (**L**) or normal (*n* = 3 three patients) (**O**) synovium. Col2 (red: **D**, **G**, **J**, **M**, **P**) and HNA (blue: **E**, **H**, **K**, **N**, **Q**) staining within each group is also presented. Note: The cell lines injected into mice shown in images I and L were derived from the same patient, but different biopsies. Tissue cytometry was employed to quantify the number of Collagen 2 positive cells in the injury site (**R**) and the HNA positive cells within the injury site and adjacent synovium (**S**). Scale bar equals 75 µm. n.s. = not significant; *p < 0.05

To determine if these synovial MPC populations have the ability to affect cartilage repair, we employed a full thickness cartilage defect (FTCD) model. One hundred thousand human synovial MPCs were intraarticularly injected into the knee joint of the NSG mice one week post-FTCD (Fig. 3A). All injuries were graded using a standardized cartilage repair score [29] and it was found that transplantation of either normal or ACL-I (non-inflamed) MPC populations resulted in increased cartilage repair post-injury; while transplantation of ACL-I (inflamed) resulted in poorer cartilage repair versus the sham (PBS) treated mice (Fig. 3B). Representative Safranin O images of the FTCD site are shown for each group (Fig. 3 C,F,I,L,O) demonstrating Safranin O positive staining within the injury site of ACL-I (non-inflamed) and normal MPCs treated mice (Fig. 3L, O) which was lacking in sham (PBS) and ACL-I (inflamed) treated groups (Fig. 3F, I). Furthermore, we observed an increased fibrotic-like response in mice that received ACL-I (inflamed), which contributed to the lower score. Collagen 2 (Col2) and human nuclear antigen (HNA) staining was undertaken to determine the impact of the MPCs on regeneration of the articular cartilage and localization of the transplanted cells, respectively. In uninjured mice, robust Col2 staining can be observed within the cartilage and no HNA present (Fig. 3D, E). In mice receiving injury but only a sham treatment (PBS), Col2 staining is disrupted/absent in the injury site and no HNA staining is present (Fig. 3G, H). When ACL-I (Inflamed) MPCs were injected, minimal Col2 staining was observed in the C site (Fig. 3J) and HNA staining was robust in the fibrotic-like lesions within the subchondral bone (Fig. 3K). This is in contrast to when ACL-I (non-inflamed) MPCs were injected in where robust Col2 staining (Fig. 3M) and minimal HNA staining was observed within the injury (Fig. 3N). When normal MPCs were injected, we observed a near complete restoration of the Col2 matrix within the injury site (Fig. 3P). Again, however, only minimal HNA staining was found within the defect site (Fig. 3Q). Instead, the synovium adjacent to the injury was almost completely comprised of HNA positive cells (Fig. 3Q).

### Quantitative proteomic analysis of MPC populations

**Fig. 4:**
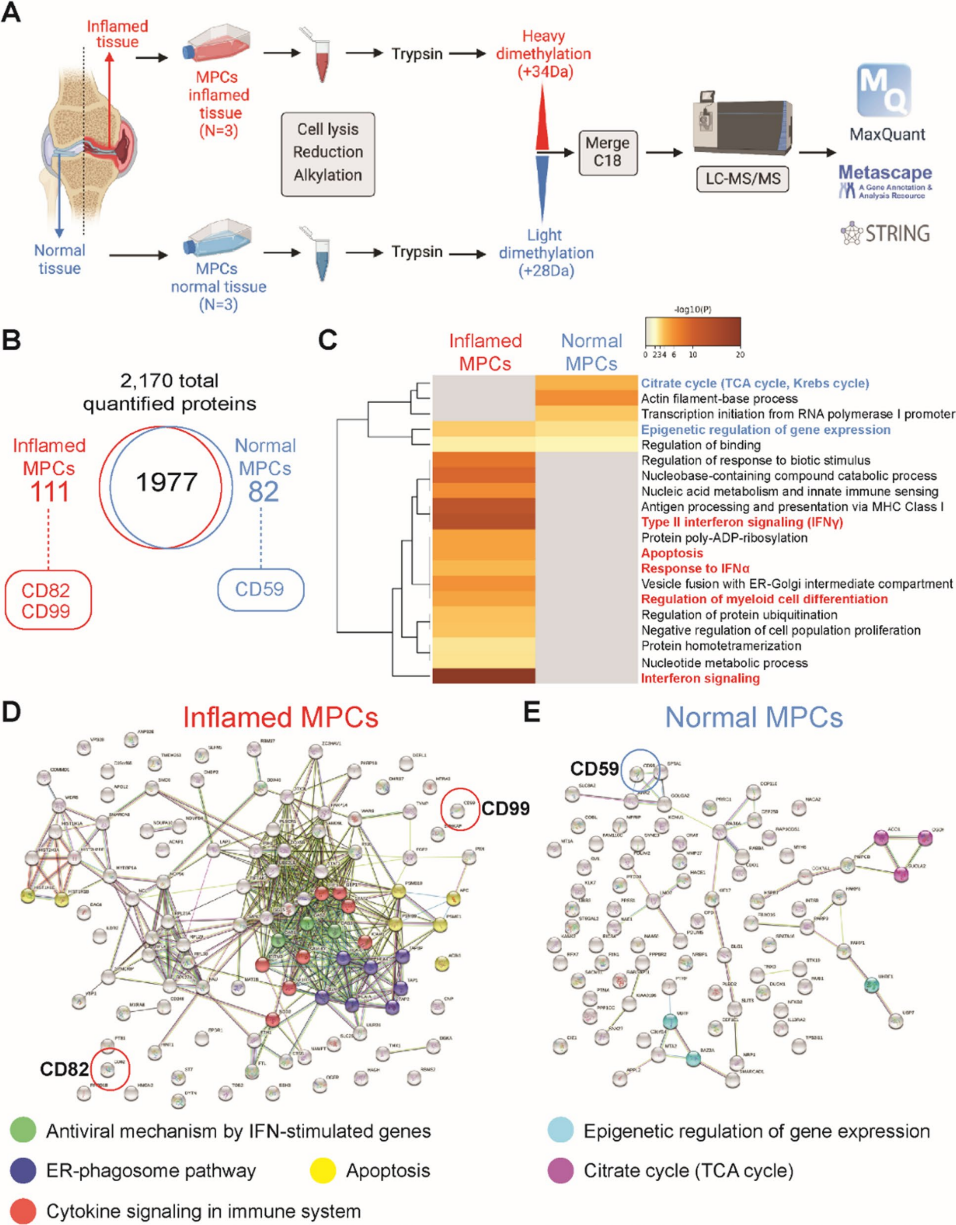
Proteomics analysis of human MPC populations from ACL-I joints. Schematic representation of the quantitative shotgun proteomics workflow (**A**). Distribution of proteins identified in each population (**B**). Heat map showing pathways regulated by differentially expressed proteins in MPCs from inflamed versus normal synovium generated with Metascape (**C**). STRING-db analysis of protein–protein interaction networks with elevated proteins in MPCs from inflamed (**D**) or normal (**E**) synovium. Colored lines between the proteins indicate different types of interaction evidence: curated databases (teal); experimentally determined interactions (pink); predicted interactions gene neighborhood (green); gene fusions (red); gene co-occurrence (blue); text-mining (yellow); co-expression (black); protein homology (purple). MPC lines derived from *n* = 3 patients

Since we observed such a dramatic difference in cartilage repair post-FTCD with the different MPC populations, an unbiased quantitative proteomics approach was undertaken to examine the differences between ACL-I non-inflamed versus inflamed MPCs (Fig. 4A). In total 2170 proteins were quantified with the majority of these proteins [1977] being equally expressed in normal versus inflamed ACL-I MPCs (Additional file 1: Table S1). There were 111 proteins that were enriched in inflamed MPCs and 82 proteins enriched in normal MPCs (Fig. 4B). Pathway analysis was performed using Metascape [36] to determine if these changes in protein expression may trigger phenotypic differences and it was found that the inflamed MPCs demonstrated enrichment of pathways that are involved in the inflammatory response and apoptosis, while the normal MPCs shown enrichment for pathways involved in cell metabolism and gene expression (Fig. 4C–E). We also mined the proteomics data to determine if there were any cell surface markers that were differentially regulated between the populations. CD82 and CD99 were enriched in MPCs from inflamed synovium, while CD59 was enriched in MPCs from normal synovium.

### Isolation and transplantation of purified synovial MPC sub-populations

**Fig. 5:**
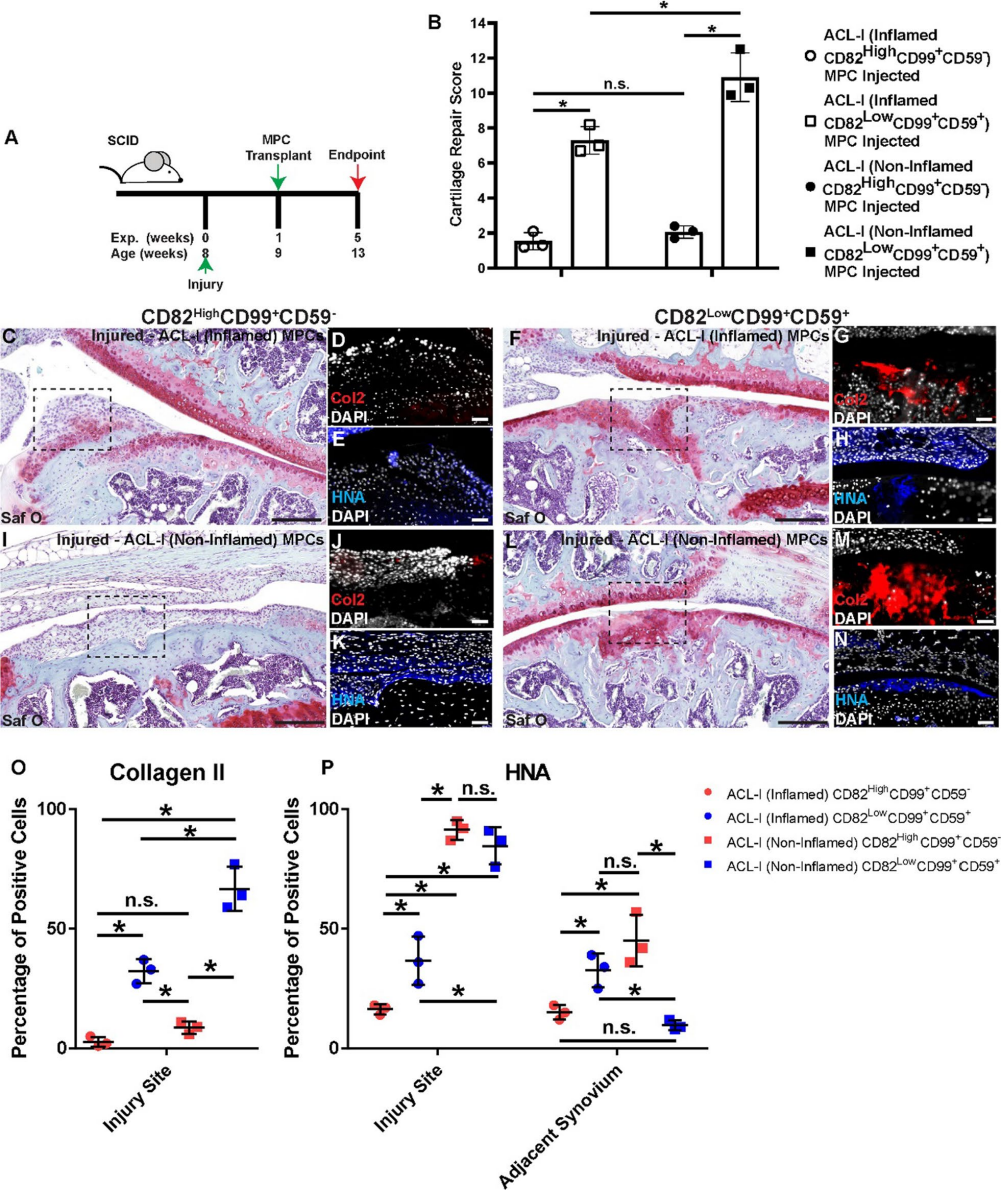
Transplanted sub-populations of human MPCs. Synovial MPCs from ACL-I patients (both inflamed (*N* = 3) and non-inflamed (*N* = 3)) were purified based on the expression of CD82 and CD59 and injected in NGS mice with a FTCD (**A**). Cartilage repair across all groups with CD82^High^CD99^+^CD59^−^ or CD82^Low^CD99^+^CD59^+^ MPCs were quantified (**B**). Safranin O staining of mice given CD82^High^CD99^+^CD59^−^ (C,I) or CD82^Low^CD99^+^CD59^+^ (**F**, **L**) MPCs. Col2 (red: D,G,J,M) and HNA (blue: **E**, **H**, **K**, **N**) staining within each group is also presented. Note: The cell lines injected in C versus F and I versus L were derived from the same biopsy. Tissue cytometry was employed to quantify the number of Collagen 2 positive cells in the injury site (**O**) and the HNA positive cells within the injury site and adjacent synovium (**P**). Scale bar equals 75 µm. n.s. = not significant; *p < 0.05

To determine if these putative ACL-I inflamed versus normal MPC markers could distinguish between cells that induce cartilage repair in vivo versus those that do not, FACS was employed to purify sub-populations of within every ACL-I (inflamed and non-inflamed) MPC line derived in the study (*n* = 19 patients, *n* = 1 inflamed and *n* = 1 non-inflamed from each of these, *n* = 1 CD82^High^CD99^+^CD59^−^ and *n* = 1 CD82^Low^CD99^+^CD59^+^ from each of those) (Additional file 2: Fig. S1). Even though our proteomics analysis suggested that CD99 was enriched in the ACL-I (inflamed) MPCs, we found that all synovial MPCs, including those derived from normal joints expressed CD99 (Additional file 2: Fig. S1C). In agreement with the proteomics data, ACL-I (inflamed) MPCs were enriched for CD82 expression versus ACL-I (non-inflamed) and normal MPCs. It was also observed that CD59 was enriched in the ACL-I (non-inflamed) and normal vs ACL-I (inflamed) MPCs populations (Additional file 2: Fig. S1D). Therefore, the CD82^High^CD99^+^CD59^−^ and CD82^Low^CD99^+^CD59^+^ sub-populations from *n* = 3 ACL-I inflamed and *n* = 3 ACL-I non-inflamed were transplanted into the FTCD model (Fig. 5).

When the CD82 ^High^CD99^+^CD59^−^ versus CD82^Low^CD99^+^CD59^+^ sub-populations were transplanted into the FTCD model, a dramatic difference was observed in the ability of these sub-populations to affect cartilage repair (Fig. 5 A). Specifically, the mice treated with the CD82^Low^CD99^+^CD59^+^ population demonstrated robust cartilage repair while the animals receiving CD82^High^CD99^+^CD59^−^ MPCs showed little to no cartilage repair (regardless of the parental MPC group)(Fig. 5B). This is clear by the representative safranin O images. In mice receiving CD82^High^CD99^+^CD59^−^ MPCs, a potent fibrotic response was observed in the injury site (Fig. 5I) and some mice even developed what appeared to be osteophytes at the injury site (Fig. 5C). In animals receiving the CD82^Low^CD99^+^CD59^+^ population, a robust chondrogenic response was observed within the defect site (Fig. 5F, L). When the Col2 staining was undertaken to identify if regeneration of a native-like ECM occurred, it was found that little to no Col2 was observed within the injury site of animals treated with CD82^High^CD99^+^CD59^−^ MPCs (Fig. 5D, J), however, Col2 staining was observed in the injury site of animals treated with CD82^Low^CD99^+^CD59^+^ MPCs (Fig. 5G, M). When the localization of the human MPCs was examined through HNA expression, it was observed that the CD82^High^CD99^+^CD59^−^ MPCs localized to fibrotic-like tissue within the injury site, but interestingly, were not found with osteophyte-like protrusions (Fig. 5E, K). In the animals receiving CD82^Low^CD99^+^CD59^+^ MPCs, HNA staining was observed in the injury site, but also was enriched in the adjacent synovium (Fig. 5H, N).

The number of Col2 or HNA positive cells were quantified, and it was observed that there were significantly more Col2 positive (presumptive chondrocytes) within the FTCD site of mice treated with CD82^Low^CD99^+^CD59^+^ MPCs regardless of the patient population the cells came from - ACL-I inflamed versus non-inflamed (Fig. 5O). However, there were also more Col2 positive cells in animals treated with CD82^Low^CD99^+^CD59^+^ MPCs derived from ACL-I non-inflamed versus inflamed (Fig. 5O). In terms of transplanted cells, we observed significantly more human MPCs in the FTCD site when the cells were derived from ACL-I non-inflamed versus inflamed regardless of cell surface marker expression (Fig. 5P), while no discernible trend was identified in the adjacent synovium.

### Isolation and transplantation of purified synovial MPC sub-populations from normal joints

**Fig. 6:**
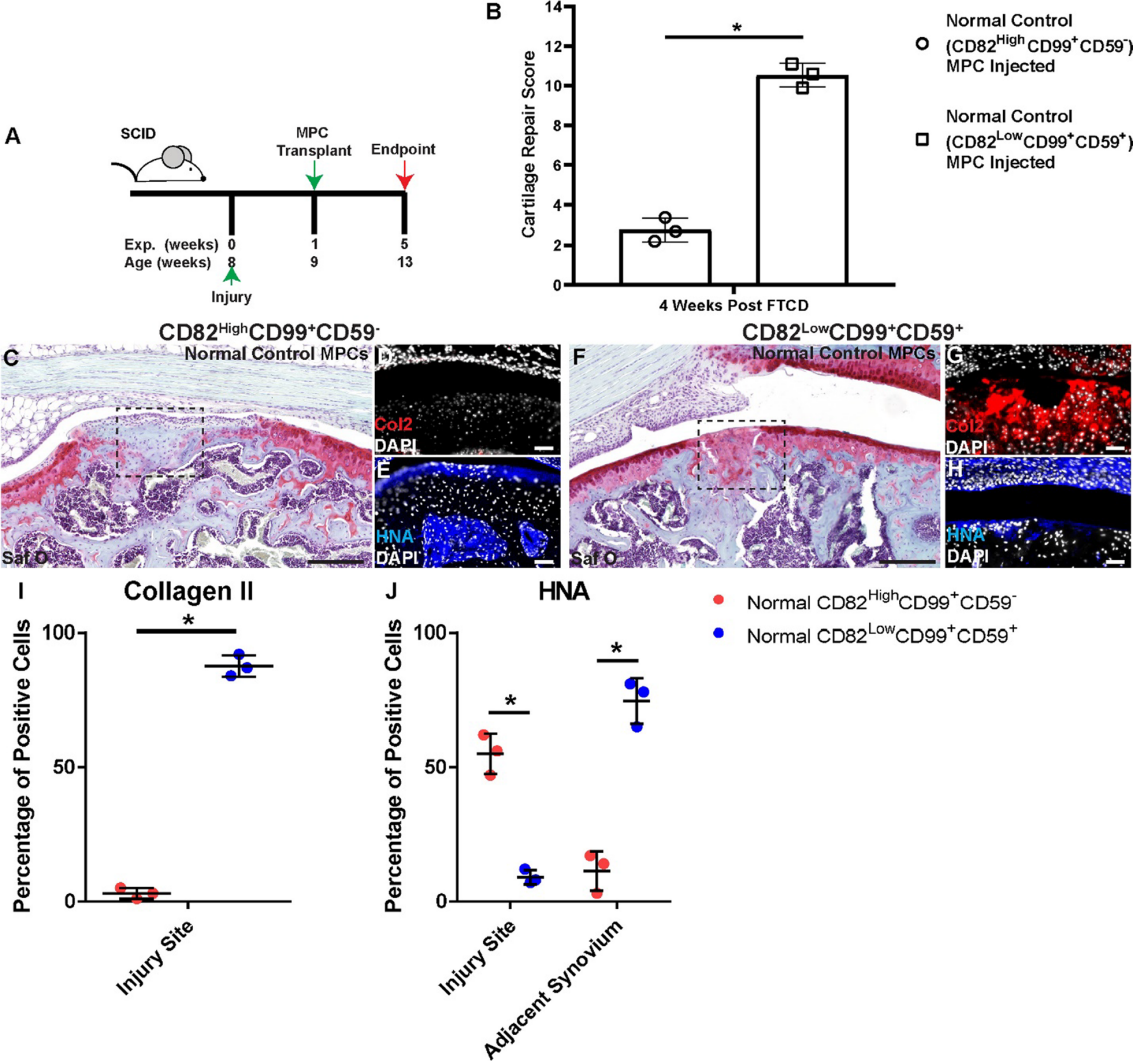
Transplanted MPC sub-populations from normal synovium. MPCs from normal synovium were purified based on the expression of CD82 and CD59 and injected in NGS mice with a FTCD (A). Cartilage repair across all groups with CD82^High^CD99^+^CD59^−^ or CD82^Low^CD99^+^CD59^+^ MPCs were quantified (**B**). Safranin O staining of mice given CD82^High^CD99^+^CD59^−^ (**C**, **I**) or CD82^Low^CD99^+^CD59^+^ (**F**, **L**) MPCs. Col2 (red **D**, **G**, **J**, **M**) and HNA (blue **E**, **H**, **K**, **N**) staining within each group is also presented. Note: The cell lines injected in C versus F were derived from the same biopsy. Tissue cytometry was employed to quantify the number of Collagen 2 positive cells in the injury site (**I**) and the HNA positive cells within the injury site and adjacent synovium (**J**). Scale bar equals 75 µm. n.s. = not significant; *p < 0.05

Since it was possible to isolate MPCs from ACL-I synovium that demonstrated the ability to promote cartilage repair, we next asked if the same approach could be employed with synovium from normal joints (Fig. 6A). When we compared the CD82^High^CD99^+^CD59^−^ versus CD82^Low^CD99^+^CD59^+^ MPC sub-populations derived from normal synovium (*n* = 3 patients), we clearly observed a reduction in repair in animals given CD82^High^CD99^+^CD59^−^ MPCs and an increase in repair in animals given CD82^Low^CD99^+^CD59^+^ MPCs (Fig. 6B). This is also evident from the Safranin O staining, in where animals receiving the CD82^High^CD99^+^CD59^−^ demonstrated a robust fibrotic-like response (Fig. 6C) and animals receiving CD82^Low^CD99^+^CD59^+^ displayed near complete cartilage regeneration (Fig. 6F). This observation was confirmed by the Col2 staining with a continuous band of Col2 staining observed in the cartilage of mice that received CD82^Low^CD99^+^CD59^+^ MPCs (Fig. 6 G). When the HNA expression was examined, it was observed that CD82^Low^CD99^+^CD59^+^ MPCs were found within the fibrotic-like tissue (Fig. 6E), while minimal CD82^Low^CD99^+^CD59^+^ MPCs were observed in the injury site but were instead enriched in the adjacent synovium (Fig. 6H).

The number of Col2 or HNA positive cells were again quantified in these animals, and it was observed that there were significantly more Col2 positive (presumptive chondrocytes) within the FTCD site of mice treated with CD82^Low^CD99^+^CD59^+^ MPCs (Fig. 5I). In terms of transplanted cells, we observed significantly more CD82^Low^CD99^+^CD59^+^ MPCs in the adjacent synovium and increased numbers of CD82^High^CD99^+^CD59^−^ within the defect site (Fig. 5J).

### Differentiation potential of synovial MPC sub-populations

Since we observed a dramatic difference in the ability of CD82^High^CD99^+^CD59^−^ versus CD82^Low^CD99^+^CD59^+^ MPCs to induce cartilage regeneration in vivo, it was decided to examine the multipotent differentiation potential of the two sub-types within each patient group (and all patients in the study). There were no differences in the expression of *ADIPOQ* or *PPARγ* post-adipogenic induction between any of the sub-populations in any of the groups with all sub-populations retaining the ability to effectively undergo adipogenic differentiation (Additional file 2: Fig. S2). However, when the osteogenic (*SP7, RUNX2*) and chondrogenic (*SOX9*, *ACAN*, *COL2A1*) markers were examined, the CD82^High^CD99^+^CD59^−^ sub-population showed a dramatic reduction in expression regardless of the group (ACL-I inflamed/ non-inflamed, normal) that the MPCs were derived from (Fig. S2).

### *Identification of synovial MPC sub-populations *in vivo

**Fig. 7:**
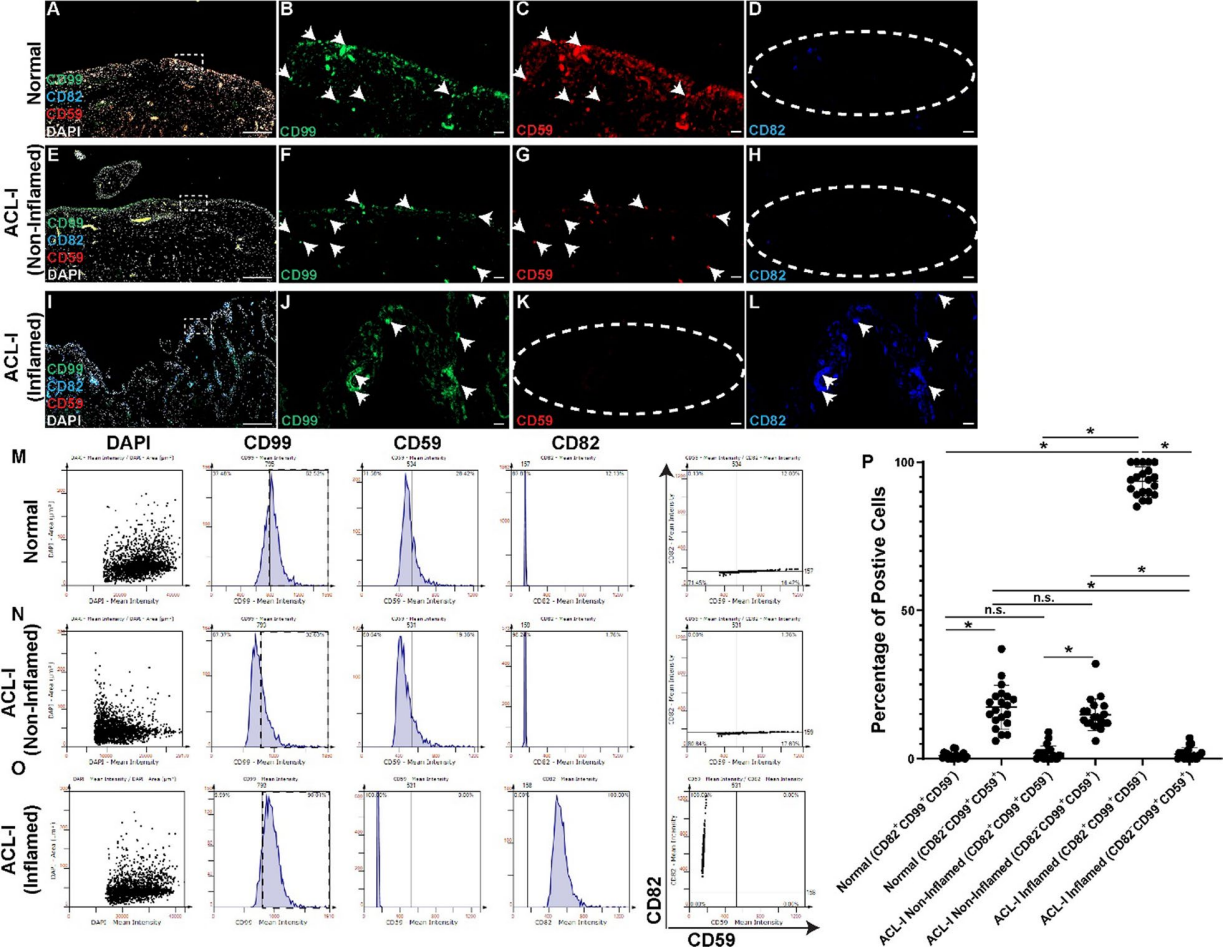
Identification and quantification of MPCs sub-populations in vivo. Representative images of CD99, CD59, CD82 stained synovium from normal (*n* = 5, **A**–**D**), ACL-I (non-inflamed, *n* = 19) (**E**–**H**) and ACL-I (inflamed, *n* = 19) (**I**–**L**). Representative tissue cytometry gates from the same groups (**M**–**O**). Quantification of the tissue cytometry results (**P**). n.s. = not significant; *p < 0.05. Arrow heads show examples of positive staining while dashed ellipses show absence of staining for that specific marker

Since these sub-populations of synovial MPCs have not been previously reported, we examined if cells expressing these markers were present in the synovium in vivo. The synovial biopsies from all normal joints and patients and ACL-I (non-inflamed and inflamed) groups were stained for CD99, CD82 and CD59 (Fig. 7A-L). CD99 was observed throughout the synovium (intima and sub-intima) in all samples (Fig. 7B, F, J). CD59 expression was enriched in normal and ACL-I (non-inflamed) synovium, with only minimal staining in the ACL-I (inflamed) synovium (Fig. 7C, G, K). When expression of CD82 was examined, few to no positive cells were identified in normal and ACL-I (non-inflamed) synovium (Fig. 7D, H), however, most cells in the ACL-I (inflamed) synovium appeared to express CD82 (Fig. 7L). To quantify these results, a tissue cytometry approach was employed, and representative gates are shown for each group (Fig. 7 M–O). When these results were analyzed, it was clear that CD82^Low^CD99^+^CD59^+^ are enriched in normal and ACL-I (non-inflamed) synovium, while CD82^High^CD99^+^CD59^−^ MPCs are enriched in ACL-I (inflamed) synovium (Fig. 7P).

## Discussion

Since the 1700’s, it has been observed that cartilage has minimal intrinsic regeneration capacity with [37] Heinemeier et al., conclusively demonstrating that mature articular cartilage does not maintain nor replace itself in normal or OA joints [38]. This study reinforced centuries of clinical observation, demonstrating that cartilage possesses limited repair/maintenance ability. Whereas many other tissues in the body react to injury with characteristic phasic responses of necrosis, inflammation and repair; the lack of blood supply in cartilage limits the “healing” of even partial thickness defects that can occur during injury to the joint [18, 24, 39], such as a ligament tear. It is hypothesized that even the smallest of cartilage defects cannot re-establish their essential low friction surface and over time this will result is cartilage degeneration and likely progress to OA [40–42]. In Canada, the prevalence of OA is estimated to double (to 1 in 4) by 2040, with the majority of patients undergoing symptom management until they reach an appropriate age or severity of disease for joint replacement [43]. While there is no clinical evidence that early treatment can stop, slow or reverse OA, there is a plethora of pre-clinical studies demonstrating that OA (secondary to injury) is most effectively treated in the earliest stages of the disease [4, 41, 44]. Therefore, it is essential to develop novel therapeutic approaches to enhance cartilage repair after injury, and in the case of cell therapies, it is paramount to determine which cells are most therapeutically appropriate through rigorous functional characterization and validation. It is our argument, that to date, this has not been done as we are just starting to comprehend MPC heterogeneity [10, 13, 23, 45–47] and how this might affect their clinical efficacy. Therefore, in the current study we examined synovium derived MPCs from normal and injured (ACL-I) joints to determine if MPCs from injured joints retained the ability to affect cartilage repair. Furthermore, we examined how MPC potential was impacted by synovitis and developed a robust cell surface marker profile to enrich for MPCs capable of inducing cartilage repair (in vivo) regardless of the injury state of the joint and/or level of synovitis.

This cell surface marker profile consisted of CD99, CD82 and CD59. Although CD99 expression was enriched in MPCs from inflamed synovium, we found that it was a common MPC marker in all patients. CD99 is a heavily O-glycosylated transmembrane protein that is expressed in thymocytes, plasma cells, cells within the reproductive organs (granulosa cells of the ovary, Sertoli cells of the testis) [48] and pancreatic islet cells [49]. CD99 has been shown to play roles in cell migration, cell adhesion, and differentiation of T cells [50]. CD99 is also expressed on MPCs derived from healthy tissue versus those from patients with Ewing Sarcoma [51]. Furthermore, in bone marrow derived MSCs, CD99 levels are increased when the cells are exposed to stress and this can lead to autophagy [52]. Since the joint is considered a low nutrient and low oxygen environment compared to many other tissues [53], it is possible that all of the synovial MPCs express CD99 as a survival mechanism. Since the functions of CD99 are still relatively unknown, it would be interesting to perform a mechanistic study on this marker and how de-regulating it expression impacts the behavior of the cells. CD82 is a member of the tetraspanin/transmembrane 4 superfamily and was first identified in the T cell activation process [54]. A number of studies have implicated CD82 as a suppressor of metastasis and its loss has been identified in multiple tumor types [55, 56]. It has been shown to be expressed on bone marrow MPCs, but no insights on its function has been reported in these populations [56]. CD82 has been shown to play a role in hematopoietic (HSC) [57] and muscle stem cell proliferation [58] and it thought to play a role in the HSC niche. It has also been shown to antagonize VEGF induced angiogenesis [59]. Since there is no described HSC niche within the adult synovium, it is unlikely CD82 on synovial MPCs is playing a role in that capacity. However, the inflamed synovium is known to show significant increases in blood flow and angiogenesis [60–62]. It may be possible that the MPCs in these areas are expressing CD82 to regulate this pro-angiogenic effect and it would be interesting to conditionally delete CD82 in mouse MPCs to test this hypothesis. Lastly, CD59 is a membrane anchored complement regulatory protein that inhibits the membrane attack complex (MAC) formation [63]. This prevents complement mediated cell lysis that is a hallmark of many diseases which impact the immune system. Sublytic levels of MAC formation can result in a number of changes in cell behavior such as altered proliferation and cyto/chemokine release [64]. CD59 is also known to be a marker of human HSCs, yet its role in these cells is not fully understood [65]. It has been hypothesized that the expression of CD59 on MPCs may protect them from complemented induced lysis and/or phagocytosis [66, 67]. If this is accurate, it is interesting that MPCs from normal or non-inflamed synovium show CD59 expression while it is absent in MPCs from inflamed areas. This could be interpreted as MPCs from inflamed regions being more suspectable to the immune/completement system which is activated after joint injury [68, 69].

We demonstrated that CD82 and CD59 enrich for different populations of synovial MPCs with differing abilities to affect cartilage repair in vivo, yet, it remains unknown if any/either of these markers play a functional role in that observation. A study by Bai et al., demonstrated that CD59 expression is essential for cartilage regeneration during Gecko tail regeneration and the loss of CD59 resulted in abnormal cartilaginous patterning due to decreased differentiation of blastemal cells to cartilage precursor cells [70, 71]. This suggests that CD59 marks a cell population that is essential for cartilage regeneration in species that have this innate ability, yet additional functional studies would shed light on this hypothesis. Specifically, it would be interesting to examine if forced overexpression of CD59 could increase the ability of different MPC populations to affect cartilage repair. Interestingly, CD82 and CD59 co-expression has been published in transcriptomic studies in chondroprogenitors [72], yet, to our knowledge there are no studies examining a functional role of CD82 in cartilage or chondrogenesis. Interestingly, it has been shown that cartilage invading synovial fibroblasts in rheumatoid arthritis express CD82 and it is hypothesized that CD82 expression reduces their motility once they are within the cartilage, keeping these invading cells at the site of destruction [73].

Another interesting/perplexing observation was that there was no reproducible pattern of where the transplanted cells integrated versus the healing outcome. In some cases few to no human MPCs were identified in the new cartilage tissue, yet robust cartilage regeneration was observed and vice versa. In some instances, we observed human cells within the injury site when fibrotic-like tissue was generated and human cells within the adjacent synovium when cartilage regeneration was observed. The only consistent pattern we observed was within the sub-population derived from the normal synovial MPCs in where cartilage regeneration was almost exclusively seen when the human cells integrated in the adjacent synovium and fibrotic-like repair was observed when the MPCs were found enriched in the FTCD site. It would therefore be important to increase the sample sizes of the current study so we can specifically look for phenotypic differences in the cells that may target a given population to a given tissue. A more comprehensive differentiation analysis (including histological and molecular analysis) of these populations of cells would also be beneficial to determine what pathways are differentially regulated between sub-populations and how this impacts osteo and/or chondrogenesis. Our proteomics data may hold clues to starting places to address this questions as well as potentially what kind of secreted molecules are being expressed by the CD59^+^ MPCs to induce other cells to affect cartilage repair.

## Conclusions

Overall, our data has demonstrated that standard MPC markers are not sufficient to identify synovial MPCs that can or cannot affect cartilage repair in vivo. Through the use of CD82 and CD59, a sub-population of MPCs that can induce cartilage repair can be purified from normal joints and those from patients that suffered a recent ACL injury, specifically mice transplanted with a CD82^Low^CD59^+^ population demonstrated robust articular cartilage regeneration post-injury. This work has provided the field with new cell surface marker(s) that enrich for MPCs with high regenerative potential and if employed in future clinical studies, may will increase the treatment effect of current cell therapies for joint injury and/or OA.

## Supplementary Information

**Additional file 1. Supplementary Table 1**. Shotgun proteomics proteins from messenchymal stem cells from inflammed and normal synovium of ACL injured knee joints identified without constraint by enzyme specificity rules during spectrum-to-sequence matching.

**Additional file 2. Figure S1**. Characterization and purification of sub-populations of synovial MPCs.** Figure S2**. Multipotent differ enation of CD82^High^CD99^+^CD59^-^ vs. CD82LowCD99^+^CD59^+^ MPCs.

## Data Availability

All data are available in the main text or the supplementary materials.

## Abbreviations

OA: Osteoarthritis
MPCs: Mesenchymal progenitor cells
ACL: Anterior cruciate ligament
BMI: Body mass index
IHC: Immunohistochemistry
CO_2_: Carbon dioxide
FC: Final concentration
MACS: Magnetic-activated cell sorting
FACS: Fluorescent activated cell sorting
MEM-NEAA: MEM non-essential amino acids
RT-qPCR: Reverse transcriptase quantitative polymerase chain reaction
*Sp7*: Osterix
FTCD: Full thickness cartilage defects
NSG: NOD *scid* gamma
H&E: Hematoxylin and eosin
NBF: Neutral buffered formamide
EDTA: Ethylenediaminetetraacetic
HNA: Human nuclear antigen
DAPI: 4′,6-Diamidino-2-phenylindole
HPLC: High performance liquid chromatography
MS/MS: Tandem mass spectrometry
SAMS: Southern Alberta mass spectrometry
FWHM: Full width at half maximum
AGC: Auto gain control
SD: Standard deviation
ACL-I: ACL injury
