# Supplementary material for: Mesenchymal progenitor cells from non-inflamed versus inflamed synovium post-ACL injury present with distinct phenotypes and cartilage regeneration capacity
Source: Stem Cell Res Ther. 2023 Jun 25;14:168. doi: 10.1186/s13287-023-03396-3 (PMC10291760; doi:10.1186/s13287-023-03396-3)
Supplement: Supplementary file 2 — Additional file 2. Figure S1. Characterization and purification of sub-populations of synovial MPCs. Figure S2. Multipotent differ enation of CD82HighCD99+CD59- vs. CD82LowCD99+CD59+ MPCs. [file 13287_2023_3396_MOESM2_ESM.docx]

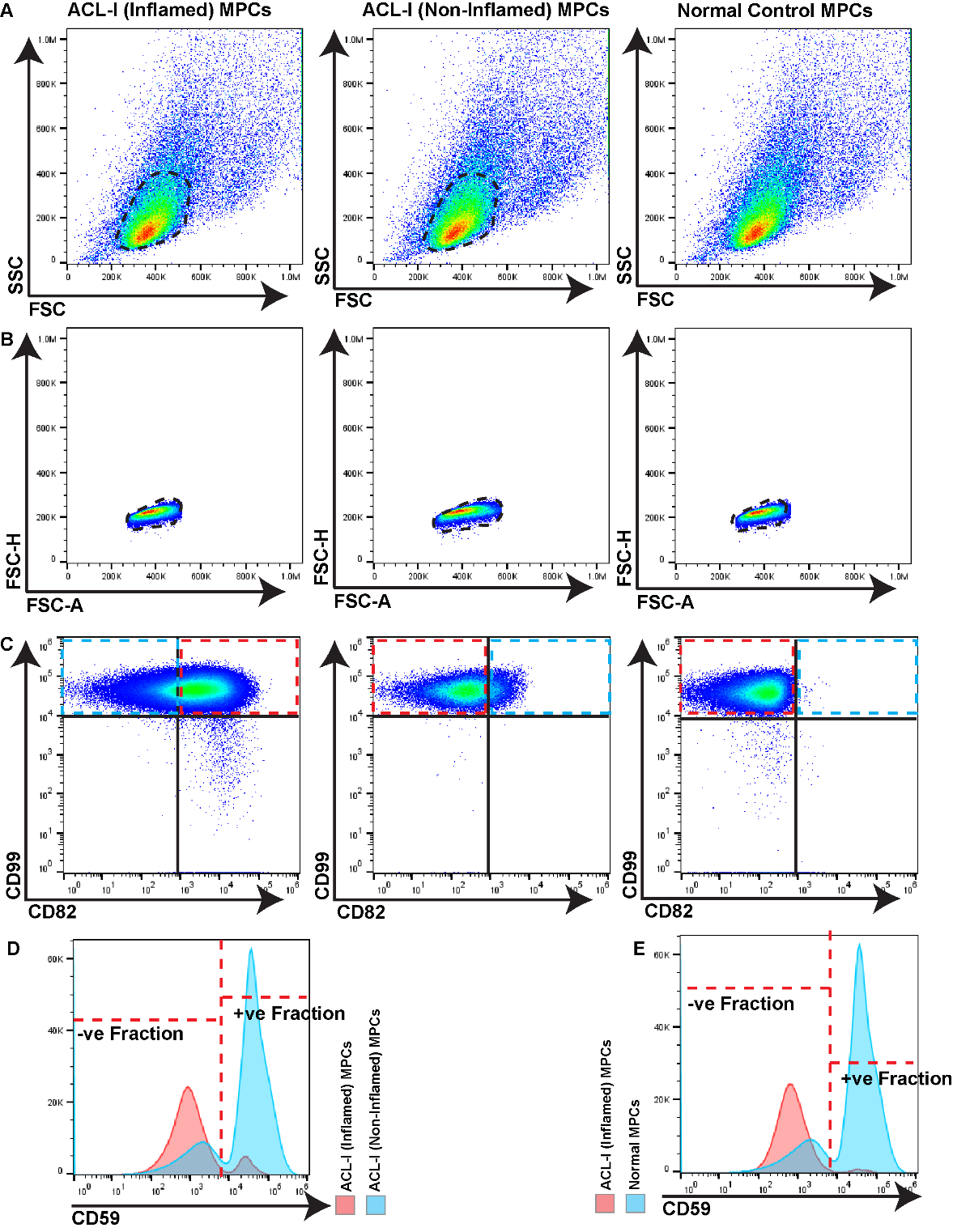


**Figure S1. Characterization and purification of sub-populations of synovial MPCs**. ACL-I (inflamed), ACL-I (non-inflamed) and normal MPCs all demonstrated similar morphology under FSC vs SSC) gating (A). The single cell populations were identified (B) and analyzed for expression of CD99 and CD82 (C). The population of interest (red or blue dashed box depending on sample (C) was then assayed for CD59 expression between ACL inflamed vs. non-inflamed (D) or ACL-inflamed vs. normal (E).


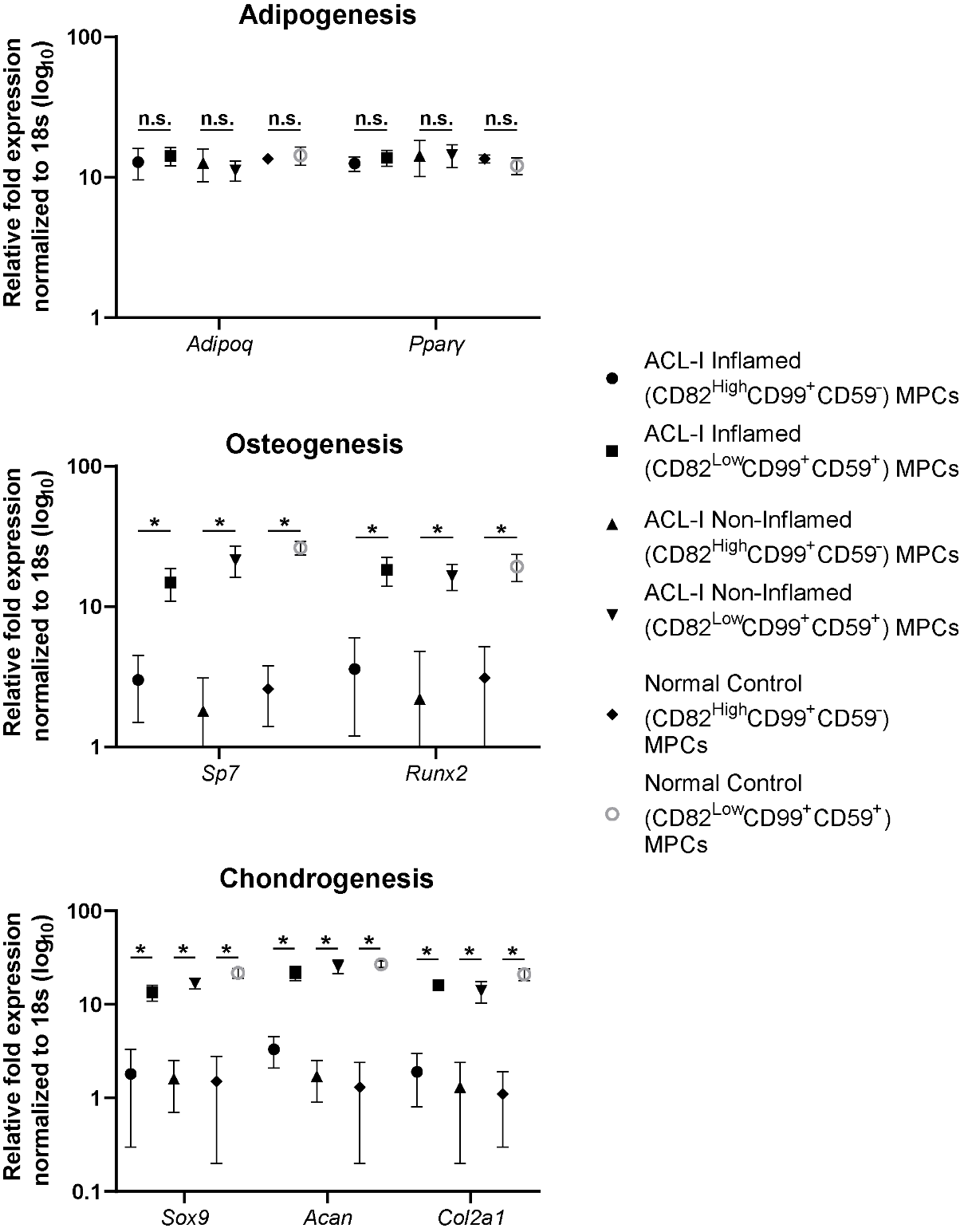


**Figure S2. Multipotent differ enation of** **CD82^High^CD99^+^CD59^-^ vs. CD82^Low^CD99^+^CD59^+^ MPCs.** Adipogenic, osteogenic and chondrogenic differentiation was assayed by qPCR expression using lineage specific markers. n.s. = not significant; * = p<0.05.
